# Suppression of Adenosine Deaminase and Xanthine Oxidase Activities by Mineralocorticoid and Glucocorticoid Receptor Blockades Restores Renal Antioxidative Barrier in Oral Contraceptive-Treated Dam

**DOI:** 10.1155/2021/9966372

**Published:** 2021-05-18

**Authors:** Olufunto O. Badmus, Emmanuel D. Areola, Eleojo Benjamin, Matthew A. Obekpa, Tolulope E. Adegoke, Oluwatobi E. Elijah, Aminu Imam, Olayemi J. Olajide, Lawrence A. Olatunji

**Affiliations:** ^1^HOPE Cardiometabolic Research Team and Department of Physiology, College of Health Sciences, University of Ilorin, Ilorin, Nigeria; ^2^Department of Public Health, Kwara State University, Malete, Nigeria; ^3^Department of Physiology, Ladoke Akintola University of Technology, Ogbomoso, Nigeria; ^4^Department of Anatomy, College of Health Sciences, University of Ilorin, Ilorin, Nigeria

## Abstract

**Objective:**

We tested the hypothesis that postpartum combined oral contraceptive (COC) treatment would induce oxidative stress via the adenosine deaminase-xanthine oxidase pathway in the kidney. We also sought to determine whether mineralocorticoid receptor (MR) or glucocorticoid receptor (GR ) blockade would suppress the activities of ADA and xanthine oxidase caused by postpartum COC treatment in the kidney.

**Methods:**

Twenty-four Wistar dams were randomly assigned to 4 groups (*n* = 6/group). Dams received vehicle (po), COC (1.0 *μ*g ethinylestradiol and 5.0 *μ*g levonorgestrel; po), COC with GR blockade (mifepristone; 80.0 mg/kg; po), and COC with MR blockade (spironolactone; 0.25 mg/kg; po) daily between 3rd and 11th week postpartum.

**Results:**

Data showed that postpartum COC caused increased plasma creatinine and urea, increased renal triglyceride/high-density lipoprotein ratio, free fatty acid accumulation, alanine aminotransferase, gamma-glutamyltransferase, uric acid, and activities of renal XO and ADA. On the other hand, postpartum COC resulted in decreased plasma albumin, renal glutathione, and Na^+^-K^+^-ATPase activity with no effect on lactate production. However, MR or GR blockade ameliorated the alterations induced by postpartum COC treatment. The present results demonstrate that MR or GR blockade ameliorates postpartum COC-induced increased activities of ADA and xanthine oxidase and restores glutathione-dependent antioxidative defense.

**Conclusion:**

These findings implicate the involvements of GR and MR in renal dysfunctions caused by COC in dams via disrupted glutathione antioxidative barrier.

## 1. Introduction

Incidence of chronic kidney disease (CKD) is increasing globally due to complications associated with the epidemic of metabolic disturbances [1]. Several factors may contribute to the progression of CKD including oxidative stress, inflammation, derangements of renal hemodynamics, and excess activation of renin–angiotensin–aldosterone system (RAAS) [2–3].

Aldosterone is a key hormone in mineralocorticoid receptor (MR) activation. Aldosterone which is also the end product of RAAS is a significant cause of renal disease [4]. Previous animal study reported that aldosterone participates in the progression of kidney disease through hemodynamic and direct cellular actions [5]. Although aldosterone is well recognized as the selective physiological ligand for MR, glucocorticoids also bind with high affinity to MR [6]. An earlier study has it that glucocorticoids are involved in the development of renal injury through a mineralocorticoid receptor-dependent mechanism [7]. Several studies have described a correlation between loss of renal function and glucocorticoid receptor (GR) activation by 11*β*-HSD enzymes [8–9]. Progression of chronic renal failure is related to excess glucocorticoid production [10].

Accumulating evidence indicates that MR and GR activation is associated with oxidative stress [11–13]. In physiological condition, there is a balance between oxidant production and antioxidant defense systems. Hence, condition of oxidative stress occurs when there is increased generation of reactive oxygen species (ROS) and/or depletion of antioxidant defense system, resulting in tissue damage. The kidney is an organ highly susceptible to damage caused by ROS. Convincing evidences have shown that increased ROS activity contributes to the pathophysiology of several kidney diseases [14–16]. Graded increase in oxidative stress markers is observed in all stages of renal disease, even in early CKD, and this could be a result of an increase in ROS as well as a decrease in antioxidant defense [17]. Adenosine deaminase (ADA) is an enzyme involved in the metabolism of purine nucleoside. It catalyzes the irreversible deamination of adenosine, an important signalling molecule that exerts antioxidant and anti-inflammatory effects, to inosine [18]. The ADA enzyme is widely distributed in tissues and plays a vital role in a numerous physiological systems. However, studies have reported that ADA activity could be used to monitor inflammation in patients with CKD [19]. Also, increased ADA positively correlates with oxidative stress [20]. Inosine is further metabolized into uric acid by the enzyme xanthine oxidase (XO), which catalyzes the last two steps of purine metabolism [21–22]. Previous studies have demonstrated that activities of XO lead to formation of superoxide anions which is the lead source of ROS production in tissues [23–24]. Moreover, earlier studies have reported a relationship between XO-induced oxidative stress and kidney dysfunction [25–26]. In the tissues, ROS levels are maintained at a physiological range by the scavenging activities of endogenous glutathione antioxidant defense mechanism [27]. Hence, in previous studies, reduction in glutathione, a vital kidney antioxidant, has been reported in CKD [28–29].

Hormone therapy such as combination of estrogen and progestin are widely used among postpartum women for both contraceptive and noncontraceptive reasons. Use of combined oral contraceptive (COC) has been shown to be associated with increased oxidative stress in premenopausal women [30]. Also, postpartum COC use elicits uric acid production that is suppressed by GR or MR blockade [31]. A previous study from our laboratory documented that COC induces hypertension that is accompanied by increased renal uric acid [32]. Likewise, combined estrogen-progestin has been linked to increased activity of renin-angiotensin system that influences blood pressure and renal function [33–35]. A previous study has demonstrated that COC activates MR through mineralocorticoid and glucocorticoid pathways [36]. Combined oral contraceptive has been reported to increase circulating glucocorticoid and aldosterone [37]. Increased glucocorticoid and aldosterone in turn activates renin-angiotensin-aldosterone system (RAAS). Activated RAAS increases generation of reactive oxygen species by activation of adenosine deaminase and xanthine oxidase [38].

Convincing evidences have shown that COC has effects on renal functions. Hence, the present study was designed to test the hypothesis that postpartum COC use would induce oxidative stress via the ADA/XO pathway in the kidney. We also sought to determine whether MR or GR blockade would suppress the activities of ADA and XO caused by postpartum COC treatment in the kidney.

## 2. Materials and Methods

### 2.1. Animals

The investigation was conducted in accordance with the National Institutes of Health Guide for the Care and Use of Laboratory Animals and was approved by the Institutional Ethical Review Board of University of Ilorin with protocol identification number UERC/ASN/2016/486. All efforts were made to minimize both the number of animals used and their suffering. Female Wistar rats weighing 130–150 g, obtained from the animal house of the College of Health Sciences, University of Ilorin, Ilorin, Nigeria, were used for the study. The animals were maintained under standard environmental conditions as follows: temperature of 24 ± 2°C, humidity of 60 ± 10%, and 12-hour dark/light cycle. Rats had unrestricted access to standard rat chow and tap water. After one week of acclimatization, rats were mated to achieve timed pregnancy at a ratio of three female Wistar rats to one male Wistar rat. The presence of spermatozoa and oestrus phase (vaginal plug) was taken as gestational day zero (0). After delivery, the pups were removed from the dams to eliminate the effects of lactation. Three weeks post delivery, twenty-four [24] dams were randomly assigned to 4 groups (*n* = 6/group).

Control dam (Dam) received distilled water (vehicle; po), COC-exposed dam (Dam+COC) received estrogen-progestin COC steroids (1.0 *μ*g ethinylestradiol EE and 5.0 *μ*g levonorgestrel LN; po), Dam+COC+GR blockade (Dam+COC+GRB) received combination of COC and glucocorticoid receptor blocker (mifepristone; 80 mg/kg; po), and Dam+COC+MR blockade (Dam+COC+MRB) received combination of COC and mineralocorticoid receptor blocker (spironolactone; 0.25 mg/kg; po) daily for eight weeks between 3rd and 11th week postpartum.

### 2.2. Sample Preparation

At the end of treatment, pentobarbital sodium (50 mg/kg, *i.p.*) was used to anesthetized the animals. Blood was collected into heparinized bottle via cardiac puncture and centrifuged at 3000 rpm for 5 minutes. Plasma was stored frozen at 4°C until needed for biochemical assay. The kidneys were excised, blotted, and weighed immediately. Following weighing, 100 mg of tissue was carefully sectioned and homogenized with a glass homogenizer. The homogenate was used for subsequent biochemical analysis.

### 2.3. Biochemical Assay

#### 2.3.1. Lipid Profile

Triglycerides (TG), total cholesterol (TC), high-density lipoprotein-cholesterol (HDL-C), and free fatty acid (FFA) were estimated in the renal tissue by standardized colorimetric methods using reagents obtained from Fortress Diagnostics Limited, Antrim, UK, following manufacturer's instruction. Ratios of TG/HDL-C and TC/HDL-C were estimated as markers of atherogenic lipid indices.

#### 2.3.2. Redox Biomarkers

Renal nitric oxide (NO) was measured by a nonenzymatic colorimetric assay kit obtained from Oxford Biomedical Research Inc., Oxford, USA, whereas renal malondialdehyde (MDA), glucose-6-phosphatedehydrogenase (G6PD), oxidized glutathione (GSSG), and reduced glutathione (GSH) were determined by standard spectrophotometric methods using reagents obtained from Fortress Diagnostics Limited, Antrim, UK. Reduced to oxidized glutathione (GSH/GSSG) ratio was estimated as an indicator of glutathione antioxidant capacity and oxidative stress. Protein levels were determined spectrophotometrically according to the Bradford method (Bradford 1976).

#### 2.3.3. Adenosine Deaminase/Xanthine Oxidase/Uric Acid Pathway

Renal adenosine and uric acid were estimated using nonenzymatic colorimetric assay kits obtained from Oxford Biomedical Research Inc., Oxford, USA, whereas activities of adenosine deaminase (ADA) and xanthine oxidase (XO) were estimated by the standard enzymatic colorimetric method using reagents obtained from Fortress Diagnostics Limited, Antrim, UK.

#### 2.3.4. Creatinine, Urea, and Albumin

Plasma creatinine, urea, and albumin were determined by standardized nonenzymatic colorimetric methods using reagents obtained from Fortress Diagnostics Limited, Antrim, UK.

#### 2.3.5. Tissue Injury Biomarkers

Renal lactate and lactate dehydrogenase (LDH) activity was measured by standardized enzymatic colorimetric method using an assay kit obtained from Fortress Diagnostics Limited, Antrim, UK. Also, gamma-glutamyltransferase (GGT), alanine transaminase (ALT), aspartate aminotransferase (AST), and alkaline phosphatase (ALP) were measured by a standardized enzymatic colorimetric method using an assay kit obtained from Fortress Diagnostics Limited, Antrim, UK.

#### 2.3.6. Na+-K+-ATPase Activity

Renal Na^+^-K^+^-ATPase activity was determined by a spectrophotometric method using reagents from Randox Laboratory Ltd. (Co. Antrim, UK).

#### 2.3.7. Statistical Analysis

Statistical analysis was performed using the SPSS software (Version 22; SPSS Inc. IL., USA), and values were expressed as mean ± SEM of 6 rats per group. One-way analysis of variance (ANOVA) was used to compare the mean values of variables among the groups. Bonferroni's post hoc test was used to identify the significance of pair wise comparison of mean values among the groups. Statistically significant differences were accepted at *p* < 0.05.

## 3. Results

### 3.1. Mineralocorticoid and Glucocorticoid Receptor Blockades Did Not Affect Kidney Weight Phenotype in Combined Oral Contraceptive-Exposed Wistar Rat Dams

**Figure 1:**
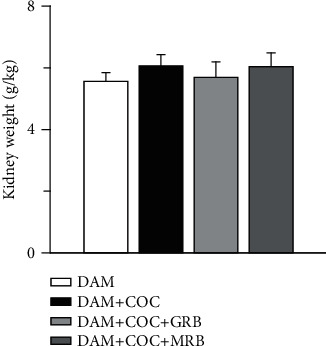
Effect of glucocorticoid (GR) and mineralocorticoid (MR) receptor blockade on kidney weight in Wistar dams exposed to postpartum combined oral contraceptives (COC). Postpartum COC treatment, GR, and MR blockade did not affect kidney weight. Post gestational COC exposure, GR and MR blockade were carried out for eight weeks after the 3-week postgestational period. Data were presented as mean ± standard error of mean, and *p* < 0.05 was taken as statistically significant. ^∗^*p* < 0.05 vs. DAM; ^#^*p* < 0.05 vs. DAM+COC. Data were analyzed by one-way ANOVA and Bonferroni's post hoc analysis. DAM: control; DAM+COC: combined oral contraceptive exposure; DAM+COC+GR: COC exposure with glucocorticoid receptor blockade; DAM+COC+MR: COC exposure with mineralocorticoid receptor blockade.

Combined oral contraceptive (COC) treatment did not change kidney weight compared with control group (Figure 1). Mineralocorticoid and glucocorticoid receptor blockades did not alter kidney weight compared with both control and animals treated with COC (Figure 1).

### 3.2. Mineralocorticoid and Glucocorticoid Receptor Blockades Altered Renal Lipid Content in Combined Oral Contraceptive-Exposed Wistar Rat Dams

**Figure 2:**
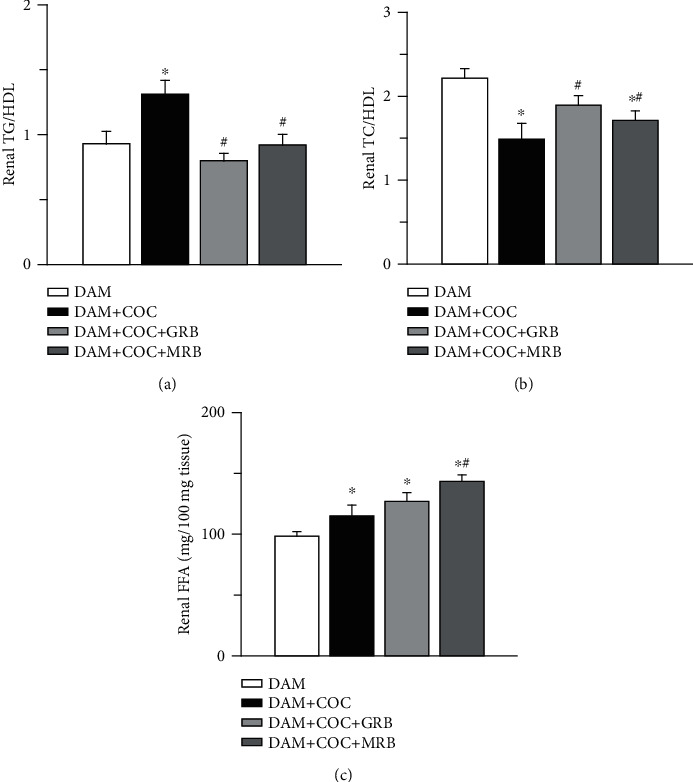
Effect of glucocorticoid (GR) and mineralocorticoid (MR) receptor blockade on renal lipid content in Wistar dams exposed to combined oral contraceptives (COC). GR and MR blockades ameliorated renal TG/HDL-C (triglyceride/high-density lipoprotein) ratio (a), MR blockade attenuated TC/HDL-C (total cholesterol/high-density lipoprotein) ratio (b), and GR and MR blockades ameliorated renal FFA (free fatty acid) (c) in COC-treated Wistar rat dams. Post gestational COC exposure, GR and MR blockade were carried out for eight weeks after the 3-week postgestational period. Data were presented as mean ± standard error of mean, and *p* < 0.05 was taken as statistically significant. ^∗^*p* < 0.05 vs. DAM; ^#^*p* < 0.05 vs. DAM+COC. Data were analyzed by one-way ANOVA and Bonferroni's post hoc analysis. DAM: control; DAM+COC: combined oral contraceptive exposure; DAM+COC+GR: COC exposure with glucocorticoid receptor blockade; DAM+COC+MR: COC exposure with mineralocorticoid receptor blockade.

There was a significant increase in renal triglyceride/high-density lipoprotein (TG/HDL) ratio and free fatty acid (FFA) but reduction in total cholesterol/high-density lipoprotein (TC/HDL) ratio in combined oral contraceptive- (COC-) treated rats compared with control (Figures 2(a)–2(c)). Glucocorticoid and mineralocorticoid receptor blockades in COC-treated dams did not alter renal TG/HDL ratio but increased renal FFA compared with control. There was reduction in TC/HDL ratio in animals exposed to postpartum COC that received MR but not GR blocker compared with control. Both MR and GR blockades in COC-treated dams reduced TG/HDL ratio and increased TC/HDL ratio compared with COC-treated dams without receptor blockade. Only MR blockade in COC-treated dams increased FFA compared with COC-treated dams without receptor blockade.

### 3.3. Mineralocorticoid and Glucocorticoid Receptor Blockades Improved Renal Glutathione-Dependent Antioxidant Defense in Combined Oral Contraceptive-Exposed Wistar Rat Dams

**Figure 3:**
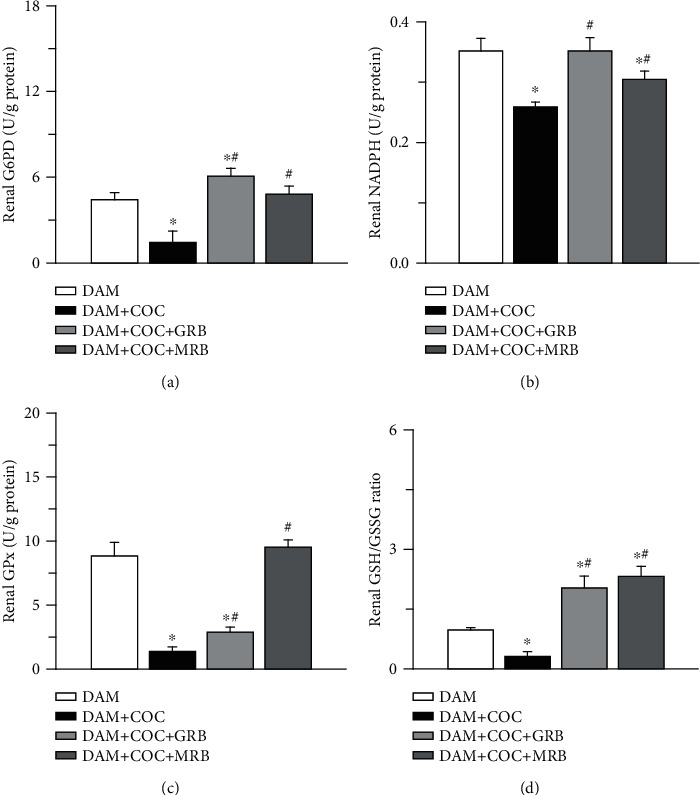
Effect of glucocorticoid (GR) and mineralocorticoid (MR) receptor blockades on glutathione-dependent antioxidant system in Wistar dams exposed to combined oral contraceptives (COC). GR and MR blockades increased renal G6PD (glucose-6-phosphate dehydrogenase) (a), increased NADPH (reduced nicotinamide adenine dinucleotide phosphate) (b), increased GPx (glutathione peroxidase) (c), and increased GSH/GSSG (reduced glutathione/oxidized glutathione) ratio (d) in COC-treated Wistar rat dams. Post gestational COC exposure, GR and MR blockades were carried out for eight weeks after the 3-week postgestational period. Data were presented as mean ± standard error of mean, and *p* < 0.05 was taken as statistically significant. ^∗^*p* < 0.05 vs. DAM; ^#^*p* < 0.05 vs. DAM+COC. Data were analyzed by one-way ANOVA and Bonferroni's post hoc analysis. DAM: control; DAM+COC: combined oral contraceptive exposure; DAM+COC+GR: COC exposure with glucocorticoid receptor blockade; DAM+COC+MR: COC exposure with mineralocorticoid receptor blockade.

Combined oral contraceptive (COC) treatment in dams resulted in reduced renal glucose-6-phosphate dehydrogenase (G6PD) activity, reduced nicotinamide adenine dinucleotide phosphate (NADPH), glutathione peroxidase (GPx) activity, and reduced glutathione/oxidized glutathione (GSH/GSSG) ratio. Renal G6PD, NADPH, GPx activity, and GSH/GSSG ratio in COC-treated dams were all increased in by MR and GR blockades compared with dam COC exposure without receptor blockade (Figure 3), but only the GSH/GSSG ratio was increased in COC-treated dams with both MR and GR blockades compared with control (Figure 3(d)).

### 3.4. Mineralocorticoid and Glucocorticoid Receptor Blockades Ameliorate Renal Lipid Peroxidation and Augmented Nitric Oxide Production in Combined Oral Contraceptive-Exposed Wistar Rat Dams

**Figure 4:**
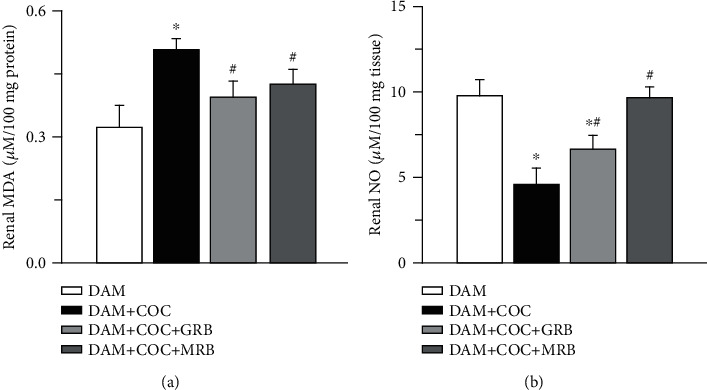
Effect of glucocorticoid (GR) and mineralocorticoid (MR) receptor blockade on renal lipid peroxidation and nitric oxide in Wistar dams exposed to combined oral contraceptives (COC). GR and MR blockade reduced renal MDA (malondialdehyde) (a) and increased renal NO (nitric oxide) (b) in COC-treated Wistar rat dams. Post gestational COC exposure, GR and MR blockades were carried out for eight weeks after the 3-week postgestational period. Data were presented as mean ± standard error of mean, and *p* < 0.05 was taken as statistically significant. ^∗^*p* < 0.05 vs. DAM; ^#^*p* < 0.05 vs. DAM+COC. Data were analyzed by one-way ANOVA and Bonferroni's post hoc analysis. DAM: control; DAM+COC: combined oral contraceptive exposure; DAM+COC+GR: COC exposure with glucocorticoid receptor blockade; DAM+COC+MR: COC exposure with mineralocorticoid receptor blockade.

Combined oral contraceptive (COC) treatment increased renal malondialdehyde (MDA) and reduced renal nitric oxide (NO) compared with control. However, MR and GR blockades reduced renal MDA and restored NO (Figures 4(a) and 4(b)) compared with COC-treated animals without receptor blockade. Compared with control, GR blockade in COC-treated animals had lower renal NO (Figure 4(b)) but not MDA (Figure 4(a)), and MR blockade in COC-treated dams had unaltered renal MDA and NO.

### 3.5. Mineralocorticoid and Glucocorticoid Receptor Blockades Altered Renal Lactate Production in Combined Oral Contraceptive-Exposed Wistar Rat Dams

**Figure 5:**
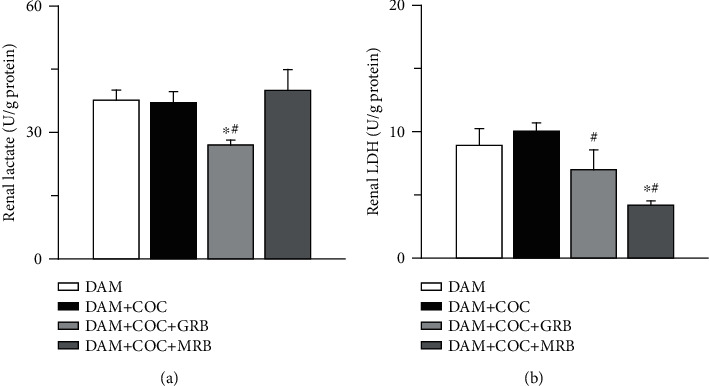
Effect of glucocorticoid (GR) and mineralocorticoid (MR) receptor blockades on renal lactate and lactate dehydrogenase in Wistar dams exposed to combined oral contraceptives (COC). GR but not MR blockade reduced renal lactate (a), and MR but not GR blockade reduced renal LDH (lactate dehydrogenase) (b) in COC-treated Wistar rat dams. Post gestational COC exposure, GR and MR blockades were carried out for eight weeks after the 3-week postgestational period. Data were presented as mean ± standard error of mean, and *p* < 0.05 was taken as statistically significant. ^∗^*p* < 0.05 vs. DAM; ^#^*p* < 0.05 vs. DAM+COC. Data were analyzed by one-way ANOVA and Bonferroni's post hoc analysis. DAM: control; DAM+COC: combined oral contraceptive exposure; DAM+COC+GR: COC exposure with glucocorticoid receptor blockade; DAM+COC+MR: COC exposure with mineralocorticoid receptor blockade.

Combined oral contraceptive (COC) did not alter both renal lactate and lactate dehydrogenase (LDH) compared with control (Figures 5(a) and 5(b)). However, GR blockade in COC-treated dams reduced renal lactate compared with both control and COC-treated dams without receptor blockade and did not alter LDH compared with control but reduced it compared with COC-treated dams without receptor blockade (Figure 5). Also, MR blockade in COC-treated dams reduced renal LDH compared with both control and COC-treated dams without receptor blockade but did not alter renal lactate but reduced LDH in COC-treated animals (Figure 5(b)).

### 3.6. Mineralocorticoid and Glucocorticoid Receptor Blockades Enhanced Renal Na^+^-K^+^ ATPase Activity in Combined Oral Contraceptive-Exposed Wistar Rat Dam

**Figure 6:**
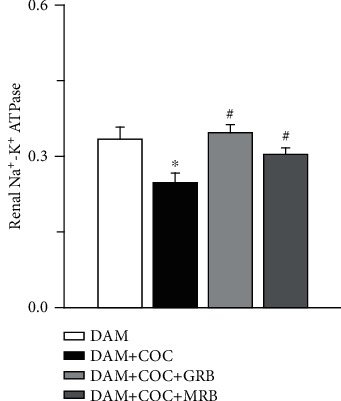
Effect of glucocorticoid (GR) and mineralocorticoid (MR) receptor blockades on renal sodium-potassium ATPase in Wistar dams exposed to combined oral contraceptives (COC). GR and MR blockades restored renal Na^+^-K^+^ ATPase (sodium-potassium ATPase) in COC-treated Wistar rat dams. Post gestational COC exposure, GR and MR blockades were carried out for eight weeks after the 3-week postgestational period. Data were presented as mean ± standard error of mean, and *p* < 0.05 was taken as statistically significant. ^∗^*p* < 0.05 vs. DAM; ^#^*p* < 0.05 vs. DAM+COC. Data were analyzed by one-way ANOVA and Bonferroni's post hoc analysis. DAM: control; DAM+COC: combined oral contraceptive exposure; DAM+COC+GR: COC exposure with glucocorticoid receptor blockade; DAM+COC+MR: COC exposure with mineralocorticoid receptor blockade.

There was significant reduction in renal Na^+^-K^+^ ATPase activity of COC-treated dams compared with control. However, MR and GR blockades in COC-treated dams increased renal Na^+^-K^+^ ATPase activity compared with dams treated with COC without receptor blockade but not control (Figure 6).

### 3.7. Mineralocorticoid and Glucocorticoid Receptor Blockades Attenuate Circulating Creatinine and Urea and Altered Albumin in Combined Oral Contraceptive-Exposed Wistar Rat Dams

**Figure 7:**
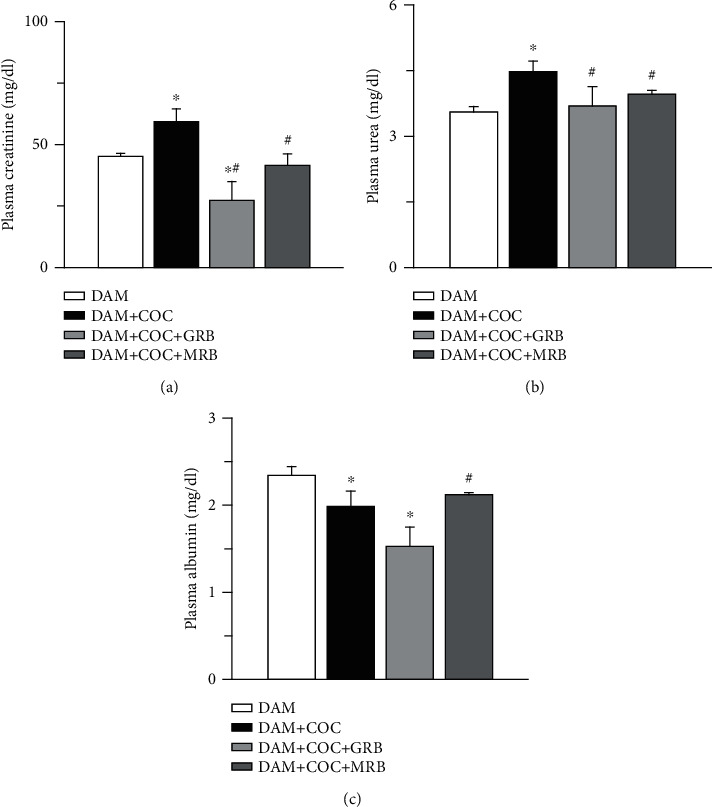
Effect of glucocorticoid (GR) and mineralocorticoid (MR) receptor blockades on plasma creatinine, urea, and albumin in Wistar dams exposed to combined oral contraceptives (COC). GR and MR blockades reduced plasma creatinine (a), GR and MR blockades reduced plasma urea (b), and GR and MR blockades increased plasma albumin (c) in COC-treated Wistar rat dams. Post gestational COC exposure, GR and MR blockades were carried out for eight weeks after the 3-week postgestational period. Data were presented as mean ± standard error of mean, and *p* < 0.05 was taken as statistically significant. ^∗^*p* < 0.05 vs. DAM; ^#^*p* < 0.05 vs. DAM+COC. Data were analyzed by one-way ANOVA and Bonferroni's post hoc analysis. DAM: control; DAM+COC: combined oral contraceptive exposure; DAM+COC+GR: COC exposure with glucocorticoid receptor blockade; DAM+COC+MR: COC exposure with mineralocorticoid receptor blockade.

Plasma creatinine and urea were elevated, but albumin was reduced in COC-treated rats compared with control. However, MR and GR blockades reduced plasma creatinine and urea compared with COC-treated dams without receptor blockade (Figure 7). Plasma creatinine and albumin were reduced in COC-treated dams with GR blockade compared with control but only MR blockade increased plasma albumin compared with COC-treated dams without receptor blockade (Figure 7(c)).

### 3.8. Mineralocorticoid and Glucocorticoid Receptor Blockades Ameliorated Tissue Injury Markers in Combined Oral Contraceptive-Exposed Wistar Rat Dams

**Figure 8:**
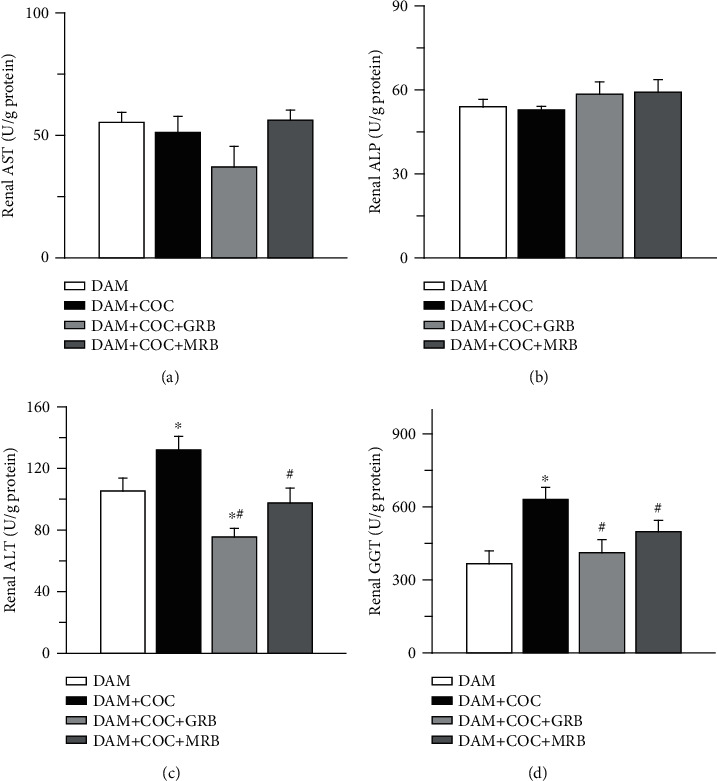
Effect of glucocorticoid (GR) and mineralocorticoid (MR) receptor blockades on renal markers of tissue injury in Wistar rat dams exposed to combined oral contraceptives (COC). GR and MR blockades did not alter renal AST (aspartate transaminase) (a) and renal ALP (alkaline phosphatase) (b), and GR and MR blockades reduced renal ALT (alanine aminotransferase) (c) and GGT (gamma-glutamyltransferase) (d) in COC-treated Wistar rat dams. Post gestational COC exposure, GR and MR blockades were carried out for eight weeks after the 3-week postgestational period. Data were presented as mean ± standard error of mean, and *p* < 0.05 was taken as statistically significant. ^∗^*p* < 0.05 vs. DAM; ^#^*p* < 0.05 vs. DAM+COC. Data were analyzed by one-way ANOVA and Bonferroni's post hoc analysis. DAM: control; DAM+COC: combined oral contraceptive exposure; DAM+COC+GR: COC exposure with glucocorticoid receptor blockade; DAM+COC+MR: COC exposure with mineralocorticoid receptor blockade.

Renal AST and ALP were not altered across the groups (Figures 8(a) and 8(b)). However, combined oral contraceptive (COC) treatment increased renal ALT and GGT compared with control. Both MR and GR blockades in COC-treated animals reduced ALT and GGT compared with those treated with COC without receptor blockade (Figures 8(c) and 8(d)).

### 3.9. Mineralocorticoid and Glucocorticoid Receptor Blockades Ameliorate Renal Uric Acid Production in Oral Contraceptive-Exposed Wistar Rat Dams

**Figure 9:**
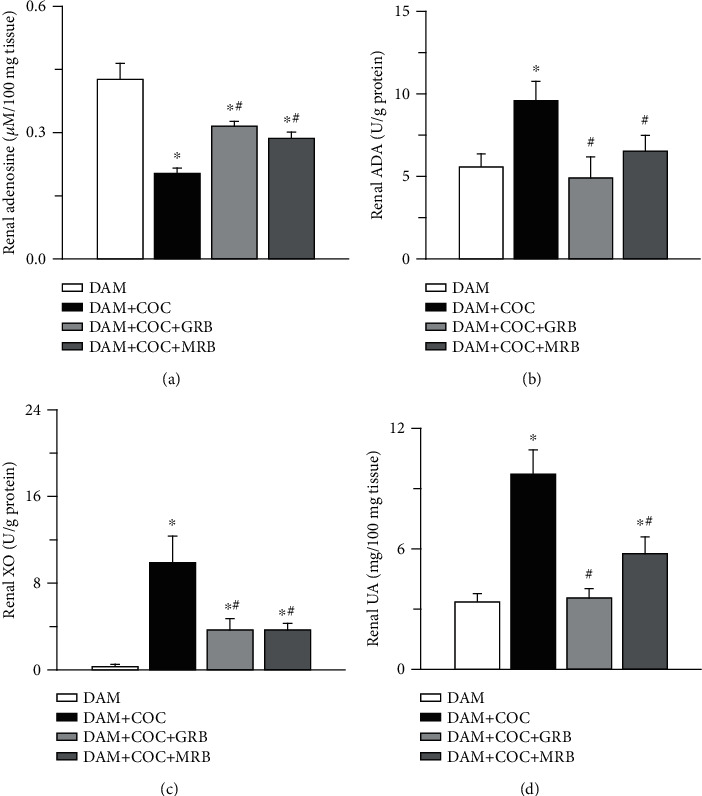
Effect of glucocorticoid (GR) and mineralocorticoid (MR) receptor blockades on renal adenosine deaminase-xanthine oxidase pathway of uric acid production in Wistar dams exposed to combined oral contraceptives (COC). GR and MR blockades increased renal adenosine (a), both GR and MR blockades reduced renal ADA (adenosine deaminase) (b), XO (xanthine oxidase) (c) and UA (uric acid) (d) in COC-treated Wistar rat dams. Post gestational COC exposure, GR and MR blockades were carried out for eight weeks after the 3-week postgestational period. Data were presented as mean ± standard error of mean, and *p* < 0.05 was taken as statistically significant. ^∗^*p* < 0.05 vs. DAM; ^#^*p* < 0.05 vs. DAM+COC. Data were analyzed by one-way ANOVA and Bonferroni's post hoc analysis. DAM: control; DAM+COC: combined oral contraceptive exposure; DAM+COC+GR: COC exposure with glucocorticoid receptor blockade; DAM+COC+MR: COC exposure with mineralocorticoid receptor blockade.

Combined oral contraceptive treatment reduced renal adenosine but increased adenosine deaminase (ADA), xanthine oxidase (XO), and uric acid (UA) compared with control. Mineralocorticoid and glucocorticoid receptor blockades increased renal adenosine but reduced renal XO compared with both control and COC-treated dams without receptor blockade and ADA compared dams treated with COC without receptor blockade but not control (Figures 9(a)–9(d)). Uric acid was reduced in COC-treated animals with either MR or GR blockade compared with COC-treated animals without receptor blockade, but only MR blockade increased renal UA compared with control (Figure 9(d)).

## 4. Discussion

In the present study, we demonstrated that postpartum COC treatment elicits elevated plasma urea and creatinine production, renal adenosine and xanthine oxidase activities, TG/HDL-C, and lipid peroxidation and disrupts nitric oxide production, Na^+^-K^+^ ATPase, and glutathione antioxidant defense in the kidney. Results from the present study further reveal that mineralocorticoid or glucocorticoid blockade attenuates urea and creatinine production, renal adenosine and xanthine oxidase activities, TG/HDL-C, and lipid peroxidation but restores renal nitric oxide, Na^+^-K^+^ ATPase, and glutathione antioxidant defense.

There is a link between lipid accumulation and renal diseases. Accumulation of lipids into nonadipose tissues such as the kidneys, an occurrence known as ectopic lipid accumulation, leads to the development and progression of renal dysfunction [39–40]. Lipids can be deposited in nearly all cell types of the kidney, ranging from mesangial cells to podocytes and proximal tubule epithelial cells, as well as damage these cells [41–42]. Excess free fatty acids (FFA) are especially detrimental to the kidneys. It can damage all the cells of the kidney via diverse means such as increased ROS production, NADPH oxidase activation, elevated lipid peroxidation, tissue inflammation, and mitochondrial damage [42–43]. A recent report has shown that administration of COC caused lipid influx into the kidneys [44]. However, in the current study, postpartum COC treatment led to increase renal TG/HDL-C ratio and FFA, suggesting that postpartum COC treatment induced lipid nephrotoxicity. On the other hand, MR or GR blockade alleviated excessive TG levels with respect to protective HDL-C, but the FFA levels remained elevated with either MR or GR treatment. There is plausibility that increased FFA in the kidney of COC-treated dams with MR or GR blockade could be a result of de novo lipolysis which breaks down TG leading to reduction in TG/HDL-C ratio compared with COC-treated animals without MR or GR blockade. The lipolytic action of MR or GR blockade could be through activation of peroxisome proliferator-activated receptor-*α* (PPAR*α*) which has been shown to enhance renal lipolysis and *β*-oxidation and combat oxidative stress [45]. Also, substances like fenofibrate that activate PPAR*α* also combat mineralocorticoid-dependent cardiac dysfunctions in animal models [46].

The kidneys are typically exposed to high levels of oxidants; for this reason, they depend on sufficient supply of glutathione to continue normal function [47]. Convincing evidence has shown that oxidative stress, resulting from an imbalance between prooxidant and antioxidant systems, largely contributes to development and progression of renal injury [15–16]. Glutathione-dependent antioxidant pathway is one of the body's endogenous means of defending the kidney from oxidative damage [28]. Glucose 6-phosphate dehydrogenase (G6PD) is the main source of nicotinamide adenine dinucleotide phosphate (NADPH) which is an essential cellular reductant in the glutathione system. NADPH is further utilized by glutathione reductase (GR) to reduce oxidized glutathione (GSSG) to reduced glutathione (GSH) [48]. Decreased G6PD activity and its resulting decreased NADPH level have been associated with diabetic kidney disease and altered nitric oxide production [49]. Nitric oxide is a vital antioxidant that plays a protective role in tissue injury, and its reduction has been linked to deficiency of G6PD activity [50]. Also, decreased intracellular antioxidants such as GPx and GSH have been reported in patients with CKD [51]. In an earlier study, decreased G6PD activity that was altered by hyperglycemia was restored by spironolactone, a MR blocker [52]. From our findings from the present study, postpartum COC treatment decreased renal NO, G6PD, NADPH, GPx, and GSH/GSSG ratio. This suggests the involvement of postpartum COC treatment in the alteration of nitric oxide and glutathione-dependent antioxidant pathway. This finding is in consonance with a recent report that showed that COC treatment reduced G6PD activity and glutathione content in female rats [44]. However, the current findings further show that MR or GR blockade restores renal nitric oxide and glutathione-dependent antioxidant pathway that was altered by postpartum COC treatment.

Disruption in the antioxidant defense system produces oxidative stress which consequently generates the inflammatory response shown by concomitant increased ADA activity [53]. ADA, an enzyme that is a mediator in the formation of some defense cells and acts as a marker of inflammation is associated with oxidative stress [54, 21, 55]. Also, it has been documented that ADA amplifies the release of toxic oxygen radicals [56]. On the other hand, ADA diminishes adenosine, a protective molecule that exerts antioxidant, anti-inflammatory properties. In the kidney, adenosine regulates renin release, glomerular filtration rate, tubular glomerular feedback, and renal vascular tone [57]. In a previous study from our laboratory, COC treatment induced hepatic ADA [58]. Our result from the present study showed that postpartum COC led to increased renal ADA and reduced adenosine. However, MR or GR blockade suppressed renal ADA and restored the renal adenosine that was altered by postpartum COC treatment. Increased levels of ADA could result in increased XO activity, which oxidizes xanthine into uric acid and concomitant generation of ROS [18]. Xanthine oxidase is a form of xanthine oxidoreductase, a house-keeping enzyme that generates ROS [24]. Elevated XO activity and its resulting increased uric acid level have been implicated in the pathogenesis and progression of ROS-induced renal diseases [26]. Malondialdehyde (MDA), a frequently used indicator of oxidative damage to tissues, is linked to uric acid production in preeclamptic women [59]. Also, overproduction of MDA resulting from increased FFA has been observed in condition of renal injury [60]. However, in the present study, postpartum COC treatment led to increased renal XO activity, uric acid, and MDA levels. Taken together, these findings from the present study imply that COC treatment after three weeks postpartum activates the ADA and XO activities with concomitant increase in uric acid production, suggesting that postpartum COC use led to ROS-induced renal tissue dysfunction. Interestingly, MR or GR blockade reversed elevated renal ADA/XO activities as well as renal uric acid and MDA production caused by postpartum COC treatment.

Impaired kidney function is indicated by elevated circulating creatinine and urea levels due to poor clearance by the kidneys. Kidney diseases such as CKD or acute kidney injury are associated with elevated plasma creatinine and urea concentrations [61]. Additionally, reduced level of circulating albumin, due to decreased production in the liver or increased loss in the kidney, is common in patients with end-stage renal disease [62]. Data from the current study that showed that postpartum COC-induced oxidative stress caused increased plasma creatinine and urea levels, but reduced albumin signifies impaired kidney function. On the other hand, MR or GR blockade restored plasma albumin and reduced elevated plasma creatinine and urea caused by postpartum COC treatment.

The current study also showed that postpartum COC-induced oxidative stress in the kidney led to increased release of tissue injury enzymes such as renal ALT and GGT; however, these were reversed by GR or MR blockade. In previous study, elevated XO activity has been linked to increase ALT and lactate production [63]. During oxygen insufficiency in the tissues, LDH, an intracellular enzyme in energy metabolism, catalyzes the conversion of pyruvate to lactate. Hence, increased tissue LDH and lactate suggest low resting oxidative capacity that has been linked with tissue injury [64]. Also, in an earlier study, elevated LDH has been used as a biomarker for early renal damage [65]. However, in the present study, postpartum COC treatment did not affect renal LDH activity and lactate production suggesting that postpartum COC-induced renal insufficiency is not associated with oxygen deficit.

Previous study has linked impaired Na^+^-K^+^-ATPase activity to renal tubular injury that was induced by elevated uric acid [66]. Also, in earlier studies, estrogen-progestin oral contraceptive treatment has been documented to reduce Na^+^-K^+^-ATPase activity in the heart and kidney [44, 67]. Similarly, in the current study, postpartum COC treatment led to impaired renal Na^+^-K^+^-ATPase activity that was restored by either MR or GR blockade.

**Figure 10:**
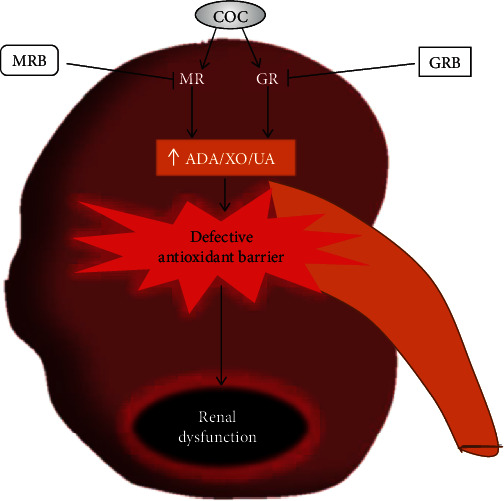
Graphical summary of the deleterious effect of COC on renal uric acid production (ADA/XO/UA) and the role of mineralocorticoid (MRB) and glucocorticoid (GRB) receptor blockades.

The present results demonstrate that depleted glutathione-induced renal dysfunction is accompanied by increased activities of renal ADA and XO in postpartum COC-treated dam. The study further shows that MR or GR blockade suppressed ADA and XO activities and restores glutathione antioxidative mechanism disrupted by postpartum COC treatment suggesting the possible involvement of GR and MR in renal dysfunction via impaired glutathione-dependent antioxidant barrier and increased ADA and XO activities (Figure 10).

## Data Availability

The numerical data used to support the findings of this study are available from the corresponding author upon request.
